# Recurrent Young Stroke With Hemianopia

**DOI:** 10.7759/cureus.38771

**Published:** 2023-05-09

**Authors:** Aparajit Ravikumar, Swathy Moorthy, Lakshmi Marappa, Emmanuel Bhaskar, Basith Ahmed

**Affiliations:** 1 Internal Medicine, Sri Ramachandra Institute of Higher Education and Research, Chennai, IND

**Keywords:** field of vision, parietal infarct, posterior circulation stroke, cerebrovascular accident, hyperhomocysteinemia, stroke

## Abstract

Homocysteine is a toxic, sulphur-containing intermediate of methionine metabolism. Hyperhomocysteinemia has been proposed as an important risk factor for ischemic stroke. We present the case of a 39-year-old male who sustained a cerebrovascular accident with left hemiparesis two years back; the patient was not compliant with his medications, and now presented with complaints of giddiness, reduced vision, and double vision. Vision disturbances were bilateral, acute in onset, progressive over time, and predominantly affected the peripheral vision. On ophthalmic examination, homonymous hemianopia was noted, and finger counting was absent in both eyes. Confrontation test revealed a bilateral reduced field of vision more so in the left eye. Baseline investigations were unremarkable except for mildly elevated serum. Homocysteine and neuroimaging showed acute infarct with hemorrhagic transformation in the right occipito-parietal region and small acute non-hemorrhagic infarcts in the right thalamus and right side of the splfingerenium of the corpus callosum. Given the visual disturbance, Humphrey visual field (HVF) perimetry was done and it revealed left homonymous congruous hemianopia, likely due to right parietal lobe infarct. The patient had recurrent infarcts previously involving anterior and posterior circulation.

## Introduction

Stroke, which is defined as the sudden onset of a neurological deficit lasting over 24 hours, is a significant cause of morbidity and mortality worldwide, presenting a considerable socioeconomic challenge in terms of stroke survivors' occupational and neuro-rehabilitation programmes [1]. While there are numerous risk factors associated with the development of stroke, much attention has been focused on homocysteine, a sulphur-containing amino acid that poses an independent risk factor for stroke separate from the well-established risk factors such as dyslipidemia, hypertension, diabetes mellitus, and smoking. Hyperhomocysteinemia has been identified as a critical risk factor for ischemic stroke [1]. According to a number of epidemiological studies, hyperhomocysteinemia may increase the risk of cerebral stroke because of its effects on venous and arterial atherosclerotic alterations, endothelial dysfunction, and vascular inflammation [2]. The World Health Organisation believes that hyperhomocysteinemia is a significant contributor to cardiovascular disease in addition to being a risk factor [2]. As a result, it has been suggested that elevated homocysteine levels be employed as a preclinical biomarker for stroke [2]. Herein, we discuss a case involving a serious visual prognosis due to a stroke resulting from elevated serum homocysteine levels.

## Case presentation

**Figure 1:**
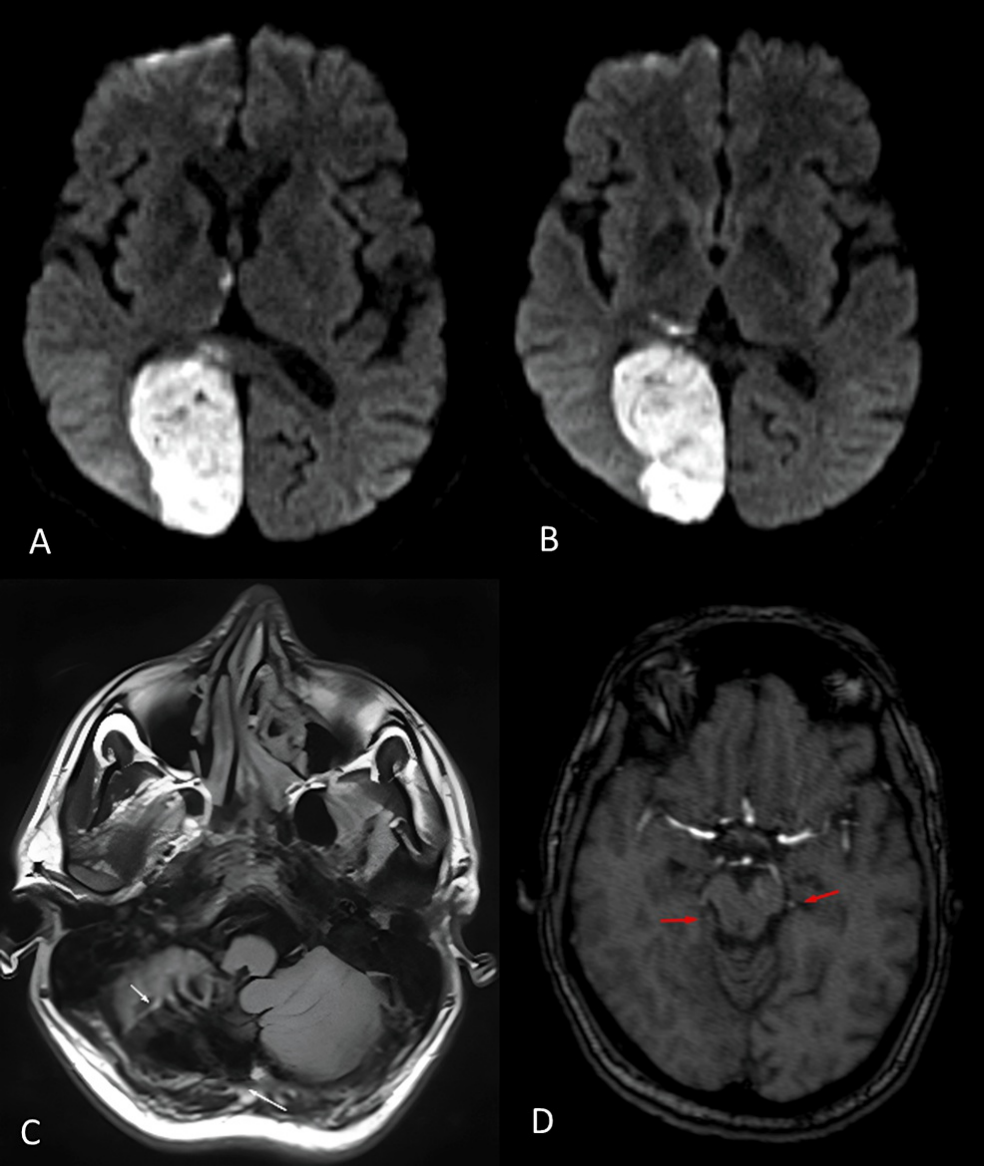
(A) Acute infarct with small areas of haemorrhagic transformation involving the right occipital lobe and a small part of adjacent parietal lobe, with small acute infarcts in the right side of splenium of corpus callosum. (B) Small acute infarcts in the dorso-medial aspect of right thalamus. (C) Chronic infarct with gliosis and hemosiderin residue in the inferior aspect of right cerebellar hemisphere. (D) Chronic infarcts with gliosis in the bilateral ganglio-capsular regions and corona radiata.

A 39-year-old male who suffered from a cerebrovascular accident two years ago and was not on regular medications presented with complaints of giddiness, gradually progressing diminution of vision for four days, and double vision in both eyes for two days. The patient did not have any other significant medical history. His vision disturbance was bilateral, with predominant peripheral vision being affected. His clinical examination revealed residual weakness in the left upper and lower limbs with mild slurring of speech. On ophthalmic examination, homozygous hemianopia was present and finger counting was absent in both eyes; the confrontation visual field test revealed that the field of vision had decreased more in the left eye. Baseline investigations revealed haemoglobin (Hb) of 9.5 gm/dL, total leukocyte count (TLC) of 10100 cells/mm3, platelet count of 6.11 cells/mm3, INR of 1.3, and glycosylated haemoglobin (HbA1c) of 7.2%. Renal and liver functions were also normal. MRI of the brain was performed which revealed an acute infarct with small areas of hemorrhagic transformation involving the right occipital and a small part of the adjacent parietal lobe, as well as small acute infarcts in the right side of the splenium of the corpus callosum and small acute infarcts in the dorso-medial aspect of the right thalamus, chronic infarct with gliosis and hemosiderin residue in the inferior aspect of right cerebellar hemisphere, chronic infarcts with gliosis in the bilateral ganglio-capsular regions and corona radiata, and severe occlusion involving P2 and P3 segments of bilateral posterior cerebral arteries (PCA) (Figure 1). 

**Figure 2:**
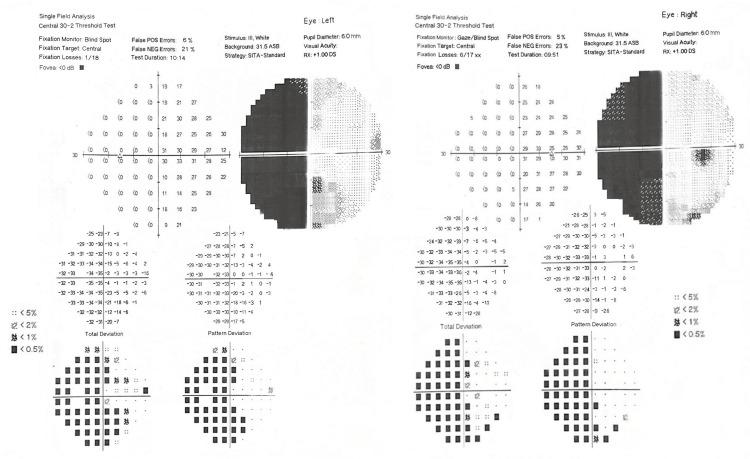
Humphrey visual field (HVF) perimetry showing left homonymous congruous hemianopia. POS - positive; NEG - negative; dB - decibel; ASB - apostilb; mm - millimeter; rx - refraction

In view of compromised visual fields revealed by the confrontation method, Humphrey visual field (HVF) perimetry revealed left homonymous congruous hemianopia, which was most likely due to a right parietal lobe infarct (Figure 2).

In view of recurrence of stroke previously involving the anterior circulation and presently the posterior circulation, the patient was restarted on dual anti-platelets (tab clopidogrel 75 mg and tab aspirin 75 mg) and statin (tab atorvastatin 40 mg) and was also started on homochek (folic acid, cyanocobalamin, and pyridoxine) due to elevated serum homocysteine levels. The patient was counselled about the poor prognosis of his vision and advised to continue the medications for life without any non-compliance. The patient is currently on regular follow-up showing mild improvement in the quality of vision but no improvement in his fields of vision.

## Discussion

With an annual mortality rate of 5.5 million people and chronic morbidity among 50% of survivors, stroke is a significant contributor to both death and long-term disability on a global scale. While bleeding in the brain or the subarachnoid space can sometimes be the cause of stroke (haemorrhagic stroke), in most cases, stroke is caused by an abrupt blockage of a blood vessel, resulting in decreased perfusion to the brain tissues (ischemic stroke) [2]. In addition to the traditional risk factors such as diabetes mellitus, hypertension, dyslipidemia, atrial fibrillation, and smoking, emerging pre-clinical studies have found that elevated circulating levels of homocysteine are an independent risk factor for systemic and ocular vaso-occlusive disorders [3].

The systemic elevation of the thiol amino acid homocysteine, which is a potentially toxic metabolite formed as an intermediate of the methionine and cystine metabolisms, is the defining feature of hyperhomocysteinemia [2]. In individuals with normal serum homocysteine levels, which typically range between 6 and 14 mol/L, a mild (15 and 30 mol/L) to moderate (30 and 100 mol/L) increase in homocysteine levels may result from a nutritional deficiency of dietary folate, vitamin B12, and vitamin B6 [2]. Hyperhomocysteinemia increases the likelihood of stroke and is mostly dependent on folic acid (vitamin B9), vitamin B12, and vitamin B6 serum levels [4]. Hyperhomocysteinemia occurs in individuals who excessively consume a diet rich in methionine and are deficient in vitamins B6, B12, and folic acid [4].

A prolonged rise in homocysteine levels triggers complex processes such as oxidative stress, calcium dysregulation, and protein homocysteinylation [4]. The occurrence of these events, combined with alterations in epigenetics, may lead to apoptosis, neuronal demise, and disruption of the blood-brain barrier, resulting in the manifestation of ischemic stroke [4]. Elevated levels of homocysteine are not only a risk factor for stroke but are also associated with a poor prognosis, the severity of stroke, and the recurrence of stroke. According to research, individuals with elevated homocysteine levels are almost twice as likely to experience a stroke compared to those with typical levels [5]. Studies indicate that a homocysteine level exceeding 10.2 mol/L can double the likelihood of stroke, and a level over 20 mol/L can increase the risk by four times [6].

The mechanisms involved in the association between plasma homocysteine levels and the risk of stroke include the following:

i. Elevated levels of homocysteine can cause oxidative damage to endothelial cells, resulting in reduced production of nitric oxide and increased adhesion of platelets to the endothelial cells. These effects can contribute to the development of thrombotic vascular disease [7,8].

ii. Elevated homocysteine levels are associated with increased platelet aggregation which occurs due to increased thromboxane A2 synthesis and decreased prostacyclin production [7].

iii. It is also associated with clotting cascade abnormalities which result in the inhibition of natural anticoagulants such as protein C and antithrombin III and the activation of factors V, X, and XII [7].

Lowering homocysteine levels below 10 mol/L is associated with a substantial reduction in the risk of stroke and decreasing levels to below 9 mol/L has been linked to a significant decrease in mortality from elevated homocysteine levels [6]. Administering folic acid, vitamins B12, and B6 in combination reduced the risk of stroke by around 25%, equivalent to an absolute risk reduction of 1.3% [9]. However, for this effect to become apparent, three years of treatment duration was required [9]. In addition to minimising functional disability days among stroke patients, this treatment has been shown to decrease the risk of non-fatal stroke [9]. Providing a daily combination of 2.5 mg folic acid, 50 mg vitamin B6, and 1 mg vitamin B12 over a five-year period resulted in a slight yet favourable impact on the prevention of stroke or fatal stroke [9]. This combination therapy thereby reduces the pathological role of homocysteine and hence lowers the vascular catastrophes of the ocular and other systems [10]. The ocular manifestations associated with hyperhomocysteinemia include papilledema, branched retinal vein occlusion, non-arteritic anterior ischemia optic neuropathy (NA-AION), temporal optic disc pallor, and field defects [10]. In the study conducted by Sowbhagya et al., hyperhomocysteinemia was associated with 14.3% of silent strokes with ocular presentations like visual field defects and optic disc pallor [10]. Vitamin therapy had a more pronounced favourable effect on the overall risk of stroke among individuals below the age of 70, those who have dyslipidemia but are not treated, and those with hyperhomocysteinemia [9]. Therefore, lowering the levels of homocysteine with supplementation of folic acid, vitamins B12, and B6, reduces the overall risk of stroke but not the severity or the disability casued by stroke [9]. Our patient was started on tab homochek (a combination of folic acid, vitamins B12, and B6) and is on regular follow-up on an outpatient basis. After restarting the stroke medications and homocysteine-lowering therapy, the quality of vision has improved, although the field of vision remains unchanged.

## Conclusions

In conclusion, regular screening for homocysteine levels in patients with stroke is essential for identifying those at risk for stroke recurrence. With the appropriate use of vitamin B12, pyridoxine, and folic acid, the levels of homocysteine in the blood can be reduced, thereby mitigating the risk of stroke recurrence. This review emphasises the significance of regular screening for homocysteine levels in all patients with stroke to help improve their quality of life and reduce the risk of further stroke-related complications.
